# Does Prosociality in Early-to Mid-Adolescence Protect Against Later Development of Antisocial Behaviours?

**DOI:** 10.1177/02724316231210254

**Published:** 2023-11-13

**Authors:** Lydia Gabriela Speyer, Ingrid Obsuth, Manuel Eisner, Denis Ribeaud, Aja Louise Murray

**Affiliations:** 1 4396 Lancaster University, UK; 2 3124 University of Edinburgh, UK; 3 2152 University of Cambridge, UK; 4 27217 University of Zurich, Switzerland

**Keywords:** antisocial behaviors, prosociality, peer problems, bullying perpetration, developmental cascades, z-proso

## Abstract

Connections between prosociality and antisocial behaviors have been recognized; however, little research has studied their developmental links longitudinally. This is important to illuminate during early adolescence as a sensitive period for social development in which prosociality could protect against the development of later antisocial behaviors. This study investigates the within-person developmental links between prosociality and antisocial behaviors, as well as a potential mediating role of peer relationships, across ages 11, 13, and 15 (*N* = 1526; 51% male) using random-intercept cross-lagged panel models. Results indicated that neither self-reported nor teacher-reported prosociality was associated with reduced aggressive behaviors but suggested a direct protective (‘promotive’) effect of teacher-reported prosociality on bullying perpetration. These findings suggest that promoting prosociality in early adolescence may help reduce some antisocial behaviors over early to mid-adolescent development. Improving prosociality could be explored as a target in intervention approaches such as school-based anti-bullying interventions.

## Introduction

Antisocial behaviors during childhood and adolescence have been associated with a large range of negative long-term outcomes including lower educational achievement, delinquency, substance use and unemployment (Miech et al., 1999; Moore et al., 2017; Otto et al., 2021; Simonoff et al., 2004), making it highly important to identify factors that can aid the prevention of such behaviors. One factor that has been suggested to have a direct protective effect on the development of antisocial behaviors is prosociality. A large amount of research has linked higher level of prosociality to lower levels of antisocial behaviors (Memmott-Elison et al., 2020). However, most studies that have explored these links have focused on exploring them in childhood up to age 12 (e.g., Eivers et al., 2012; Hay et al., 2021; Jambon et al., 2019; Kokko et al., 2006). Other research has focused on later adolescence, specifically juvenile offenders (Morales et al., 2021) or in relation to performance in high competition sports (Kavussanu et al., 2013). Far fewer studies have examined the links between prosociality and anti-social behaviors in early adolescence despite this developmental stage being crucial in young people’s social development (Blum et al., 2014). In this study, we therefore contribute to addressing this gap by examining the within-person developmental links between self- and teacher-reported prosociality and antisocial behaviors from early to middle adolescence (across ages 11, 13, and 15).

We here define prosociality as a multi-dimensional construct encompassing sympathy, empathy and prosocial behaviors. Sympathy refers to the feeling of concern or compassion for others who are experiencing negative emotions or difficulties. Empathy, on the other hand, refers to the ability to understand and share another person’s feelings and emotions, and prosocial behaviors refer to behaviors directed towards benefiting others (Hastings et al., 2007). We further define antisocial behaviors as behaviors that violate the rights of others as well as rules and norms of society (Frick & Viding, 2009). In the current study, we focus on aggressive behaviors and bullying perpetration as specific aspects of antisociality. We follow a risk and protective factor framework (Kraemer et al., 1997; Masten, 2001) to investigate whether prosociality has a direct protective effect for the development of antisocial behaviors (or equivalently, whether lower levels of prosociality act as a risk factor for the development of antisocial behaviours). Specifically, we test whether higher levels of prosociality are associated with lower levels of antisocial behaviors across early-to mid-adolescence. Within the criminology literature, this corresponds to Farrington et al.’s (2016) definition of a promotive factor.

Though historically it has often been assumed that antisociality and prosociality are opposite poles of the same construct (Wispé, 1972), research suggests that these constructs are related but distinct. They tend to emerge as separate but correlated dimensions in factor analyses (Murray et al., 2017), follow differential developmental trajectories (Eivers et al., 2010; Jambon et al., 2019), and show differential associations with other developmental predictors (Tian et al., 2018) and outcomes (e.g., Kokko et al., 2006). For instance, investigating psychological needs theory, Tian et al. (2018) found that satisfaction of competence needs at school was predictive of engaging in more self-reported prosocial but not less antisocial behaviors in school. Kokko et al. (2006) found that developmental trajectories characterized by high teacher-reported physical aggression across ages 6 to 12 were associated with increased risk of school dropout and engagement in physical violence at age 17, however, higher teacher-reported prosociality did not show a direct protective effect against these outcomes.

In acknowledgement of the idea that prosociality and antisociality are likely to be connected, a number of developmental theories have proposed that they share similar underlying mechanisms (e.g., Hastings et al., 2007; Veenstra, 2006). For example, self-determination theory suggests that engagement in prosocial and antisocial behavior is guided by different types of motivation that lie on a continuum from more externally controlled (extrinsic) motivation to more autonomous (intrinsic) motivation (Hardy et al., 2015). Research has suggested that being driven by autonomous motivation is associated with both engagement in prosocial behaviors as well as abstaining from antisocial behavior (Hardy et al., 2015).

Other studies, however, have suggested that prosociality is not necessarily negatively associated with antisocial behaviors. In fact, some studies have even found positive links between these two behaviors (Hay et al., 2021; Pakaslahti & Keltikangas-Järvinen, 2001; Veenstra, 2006). Hay et al., (2021), for instance, found that early experimentally observed prosocial behavior (sharing at age 2.5) was positively correlated with aggressive behaviors (use of force at age 2.5) whereas Pakaslahti et al. (2001) found evidence for a group of adolescents (aged 14) that was characterized by peers as both antisocial and prosocial with that group reported to be highly popular by peers. This suggests that young people may have the capacity to enact both and rely on situational factors to deploy one or the other. This is in line with the theoretical framework of framing theory (Veenstra, 2006), which suggests that young people may engage in behaviors following a gain frame, that is they may chose to engage in either prosocial or antisocial behavior depending on which behavior may lead to the highest social reward (Veenstra, 2006). Considering that adolescence is a period characterized by increased social reward sensitivity (Foulkes & Blakemore, 2016), acting in line with the gain frame may be particularly prevalent during adolescence, and has therefore also been associated with Moffitt’s (1993) definition of adolescence-limited antisocial behavior (Veenstra, 2006).

Yet, despite ample theories and studies discussing the relations between prosociality and antisocial behaviors, relatively little research has studied the developmental links between prosociality and antisocial behaviors using longitudinal designs (e.g., Chen et al., 2010; Obsuth et al., 2015). This is important because these behaviors may reciprocally influence each other over time. Specifically, while the majority of research has focused on higher prosociality as a direct protective factor for antisocial behaviors (e.g., Carlo et al., 2014; Griese & Buhs, 2014), antisocial behaviors themselves may also act as a risk factor for decreases in prosociality. For instance, acting aggressively may lead to negative feedback from peers which could result in changes in individuals’ capacity to emphasize with others or in their willingness to engage in prosocial behaviours. Children who engage in aggressive behaviors may become socially isolated and rejected by their more prosocial peers, leading them to form relationships with antisocial peers instead (Bowker et al., 2007). This not only prevents them from learning from their socially competent peers but also reinforces negative social biases, making it difficult for children to see prosocial behavior as a viable option for social gratification (Arsenio & Lemerise, 2004). Thus, potentially leading to a negative reinforcing cycle whereby decreases in prosociality lead to increases in antisocial behaviors which in turn may lead to further decreases in prosociality.

To date, very little research has investigated such reciprocal associations. Using a cross-lagged panel design, Chen et al. (2010) found that teacher-reported aggressive behaviors across grades 2 to 4 (mean age at grade 2: 8.4 years) were associated with decreased social competencies a year later, however, social competencies were not associated with changes in aggressive behaviors over time. Similarly, using a cross-lagged panel model and data from the same cohort (z-proso) as the current study albeit focusing on a younger sample, Obsuth et al. (2015) found that both teacher- and parent-reported aggressive behavior predicted decreases in teacher- and parent-reported prosociality across ages 7, 8, 9 and 11 while prosociality was not associated with changes in aggressive behaviors. Across ages 7, 8, and 9, they further found that peer difficulties mediated the links between aggressive behaviors and prosociality, suggesting that peer relationships may play an important role in connecting aggressive and prosocial behaviours across late childhood.

Obsuth et al.’s (2015) findings with respect to a mediating effect of peer relationships in the associations between aggressive behaviors and prosociality are in line with other research linking engagement in prosocial behaviors to better peer relationships (e.g., Caputi et al., 2012). Prosocial behaviors may not only help reduce the likelihood of peer rejection or bullying but may also reduce the chance of young people affiliating with antisocial peers who socialize them in antisocial behavior (Dishion et al., 1996; Veenstra & Laninga-Wijnen, 2022). Importantly, adolescents with better peer acceptance may have less to gain and more to lose from engaging in bullying and aggression, thus prosociality may reduce aggressive behaviors and bullying perpetration via increasing positive peer relationships (Zych et al., 2019). As of now, research on potential mediating mechanisms in the associations between prosociality and antisocial behaviors has been scarce (Memmott-Elison et al., 2020).

The period of early adolescence has also been neglected in longitudinal studies of the links between prosocial and antisocial behavior. This is an important gap considering that from early adolescence onwards, young people spend increasingly more time outside their family (Nelson et al., 2016), thus leading to more opportunities in engaging in prosocial and/or antisocial behaviors. Indeed according to the World Health Organisation (WHO, 2018) half of all mental health problems including conduct problems such as aggressive behaviors appear before the age of 14 years. This is perhaps not surprising as early adolescence is a dynamic developmental stage with several key milestones including the development of a the so called ‘social brain’ (Blakemore, 2012), whereby young people move from categorical, egocentric thinking to starting to develop awareness and later consideration for others. They widen their social context by developing deeper and more meaningful friendships. As such, they have more opportunities to engage in prosocial and/or antisocial behaviors, and it is therefore important to explore these during this developmental period. According to Blum and colleagues' (2014) conceptual framework of early adolescence, this developmental stage represents four core goals: engagement with learning, emotional and physical safety, positive sense of self/self-efficacy, and acquisition of life/decision skills. Despite its central role in life-course development, early adolescence as a developmental stage has been described to be understudied (e.g., Blum et al., 2014).

Research aiming to gain better insights into the developmental links between antisocial behaviors and prosociality during adolescence is particularly crucial as it can help inform prevention and interventions, especially in relation to antisocial behaviors that tend to be most problematic during adolescence such as bullying perpetration. For example, if increases in bullying perpetration are found to be preceded by lower prosociality in early adolescence, interventions may achieve a reduction in bullying perpetration by focusing on promoting prosociality. Importantly, interventions are usually targeted at the within-person level (Hamaker et al., 2015). However, most longitudinal research to date that has focused on links between antisocial behaviors and prosociality has used methods that conflate within- and between-person effects, such as the cross-lagged panel model (Chen et al., 2010; Obsuth et al., 2015). As these models do not disaggregate within-from between-person effects, these models cannot give clear insights into the within-person relations that are of primary interest for interventions (Hamaker et al., 2015).

The aim of the present study is to advance current understandings of the within-person developmental links between prosociality and antisocial behaviors, focusing primarily on whether prosociality during early adolescence may protect against the development of later antisocial behaviors. Specifically, we investigate whether within-person changes in teacher-as well as self-reported prosociality are associated with within-person changes in aggressive behaviors and bullying perpetrations and vice versa. We are taking a multi-informant perspective given that prior research has found discrepancies in reported levels of prosociality depending on the informant. For instance, a recent study on cross-informant discrepancies conducted in the here used sample found that young people report higher levels of prosociality than attributed to them by their teachers (Murray et al., 2022). To explore a potential mechanism by which prosociality and aggressive behaviors may become linked, we further investigated whether peer relationships mediated the associations between prosociality and bullying perpetration as well as aggressive behaviors. We hypothesize that higher prosociality in early adolescence protects against increases in aggressive behaviors, and bullying perpetration, whereas being a bully and engaging in aggressive behaviors will be associated with decreases in prosociality over time. Further, we hypothesize that better peer relationships mediate the associations between higher prosociality and less aggressive behaviors and bullying perpetration. Thus, following framing theory, we predict that one way by which prosociality and antisocial behaviors may become linked relates to adolescents choosing to engage in actions that result in positive peer relationships as a form of social reward.

## Methods

### Participants

Participants in this study took part in the Zurich Project on the Social Development from Childhood to Adulthood (z-proso). Starting data collection in 2004 when children entered primary school at age 7, the longitudinal cohort study z-proso has been following the lives of approximately 1500 children of an initial targe sample of 1675 children growing up in Zurich. The most recent wave of data collection was carried out in 2022 at age 24. The culturally diverse sample was recruited based on a stratified sampling design that selected 56 schools, including 116 classes, based on school size and area-based deprivation to ensure representativeness. The main data collection waves took place when children/young people were aged 7, 8, 9, 11, 13, 15, 17, and 20 with data collection still ongoing.

Originally, one of the aims of z-proso was to investigate the effect of two evidence-based intervention programs. Parents of participants in 28 schools were invited to participate in the Triple P - positive parenting program. Fourteen of these schools as well as another 14 schools took part in the PATHS (Promoting Alternative Thinking Strategies) social skills training (Ribeaud et al., 2022). Analyses of the effect of these interventions have suggested that they did not have any consistent effect on the different dimensions of child problem behaviors (Averdijk et al., 2016). Further, analyses of response shifts in teacher-reported externalising behaviors indicated that participating in the PATHS intervention did not change teacher’s reporting behavior (Murray, Booth, et al., 2019). Thus, this data is generally treated as observational data. For more information on the intervention component of z-proso, see the cohort profile (Ribeaud et al., 2022).

In the current study, we focus on the early adolescent period and use data from the age 11 (collected in 2008), 13 (collected in 2010), and 15 (collected in 2012) wave as, at these time points, the variables of interest to this study were consistently measured (e.g., teacher-reports were only available for a subset of participants at age 17 while self-reports were only available in a different format at earlier ages). Basic sample demographics are available in Table 1. Analyses of non-response in z-proso have suggested that participation and dropout were largely independent of a range of variables including aggression when adjusting for a range of other factors potentially related to drop-out such as parental education or neighborhood social class. One of these factors, that is having parents with a mother tongue other than German (Zurich’s official language) was significantly associated with higher risk of drop-out across both unadjusted and adjusted models (N. L. Eisner et al., 2019). Additional information on z-proso, including detailed information on recruitment and study procedures can be found in the cohort profile paper (Ribeaud et al., 2022) and on the study website: http://www.jacobscenter.uzh.ch/en/research/zproso/aboutus.html.

**Table 1.** Sample Demographic Information.

Variable	Category	%	N
Child sex	Female	48.6	724
	Male	51.4	784
Country of birth	Switzerland	49.1	749
Female primary caregiver	Serbia-Montenegro (incl. Kosovo)	6.8	103
	Portugal	5.8	88
	Sri Lanka	5.4	82
	Germany	3.7	57
	Italy	3.5	54
	Other	25.8	393

		Mean	SD
Child age at data collection	Age 11 wave	11.33	.37
	Age 13 wave	13.67	.36
	Age 15 wave	15.44	.36

### Ethical Approval and Consent

The study received ethical approval from the University of Zurich from the Ethics Committee from the Faculty of Arts and Social Sciences of the University of Zurich. Active informed consent was provided by parents up until age 12, after which active informed consent was obtained from the participants directly. Parents could still choose to opt their child out until the age of 18.

### Measures

Data on aggressive behaviors, and prosociality was collected at ages 11, 13, and 15, using adapted teacher-reported as well as self-reported versions of the Social Behavior Questionnaire (SBQ) (Tremblay et al., 1991). Of note, the self-report assessments generally took place a few weeks/months before the young person assessments. The SBQ measures youths’ psycho-social development across five areas including anxiety/depression, aggression, non-aggressive conduct problems, ADHD symptoms and prosociality. Behaviors were measured on a 5-point Likert-type scale ranging from *never* to *very often* and were adapted for age-appropriateness, for instance referring to adolescents rather than kids in later assessment waves. Importantly, the self-reported and the teacher-reported versions of the SBQ differed slightly in how they measured prosociality. That is, self-reported SBQs encompassed items on prosocial behaviors, sympathy, and empathy, whereas teacher-reported SBQs only captured behavior-based components of prosociality.

The self-reported SBQ versions completed at the age 11, 13, and 15 wave of z-proso included 8 prosociality items, referring to empathy (*You were good at understanding another person’s feelings*), sympathy (*You showed sympathy to someone who was upset or had hurt himself/herself*) and a variety of prosocial behaviors (e.g.*, You volunteered to help to tidy or clear up a mess*) as well as 15 aggressive behavior items covering physical aggression (e.g., *You kicked, bit, or hit someone else*), reactive aggression (e.g.*, You got very angry when someone teased or irritated you*), proactive aggression (e.g., *You intimidated someone else to get what you wanted*), oppositional aggression (e.g., *You hit or kicked your parents when you were angry*) and indirect aggression (e.g., *When you were mad at another kid you got others to dislike that kid as well*). Youth were asked to indicate how often they engaged in a certain behavior over the past 12 months.

The teacher-reported SBQ versions included 6 items referring to prosocial behaviors (e.g.*, <Child> volunteered to help to tidy or clear up a mess*) and 13 items referring to aggressive behaviors covering reactive aggression (e.g.*, <CHILD> reacts in an aggressive manner when teased*), proactive aggression (e.g., *<CHILD> scares other children to get what he\she wanted*), and physical aggression (e.g.*, <CHILD> gets into fights*). The numbers of teachers providing ratings at each measurement time were 274 at age 11, 265 at age 13, and 258 at age 15, rating 1,036, 1268 and 1287 students across 116 classrooms respectively with students not necessarily being rated by the same teacher each year. Specifically, students transitions from primary to secondary school between the age 11 and age 13 wave, thus teachers and classrooms changed across that period. Items were summed up to derive composite scores for the respective constructs. Psychometric analyses of the SBQ in the study sample have been favorable and supported the reliability, factorial validity, and measurement invariance up to the metric level (Murray, Eisner, & Ribeaud, 2019). As for the self-reported items, children were asked to indicate how often they engaged in a certain behavior over the past 12 months.

At the same ages, peer relationships were measured as part of an assessment of school functioning, including three items on adolescents’ bonds within their class (e.g., *The other adolescents in my class are nice to me*) as rated by the young people themselves. Items were scored an a 4-point scale ranging from *fully untrue* to *fully true* and subsequently summed up to derive a composite score. Of note, participants transitioned into secondary school after the age 11 wave, thus peer relationships referred to different classmates at age 11 compared to age 13 and age 15. At each time-point, participants were asked to indicate their agreements to the items referring to their experiences while attending their current school.

Bullying perpetration was measured using the self-reported Zurich Brief Bullying Scales (ZBBS) (Murray et al., 2021). At ages 11, 13, and 15, the ZBBS included one item each on engagement in verbal aggression, physical aggression, property destruction and social exclusion over the past year (e.g., *Have you purposely ignored or excluded anyone*). Items were scored on a 6-point scale ranging from *never* to *(almost) every day* and summed up to derive a composite measures for bullying perpetration. The ZBBS has been found to have reasonably good psychometric properties in the current study sample, however, it’s longitudinal measurement invariance has been noted to be limited with physical forms of bullying becoming less relevant across adolescence (Murray et al., 2021).

A complete list of all questionnaire items used in the current study is available in the Supplementary Materials Table S1. Descriptive statistics including reliability coefficients (Cronbach’s alpha) are included in Table S2.

### Statistical Analysis

To investigate the within-person developmental links between prosociality and aggressive behaviors, bullying perpetration and peer relationships, random-intercept cross-lagged panel models (RI-CLPM) were fit. RI-CLPM models combine the key features of the cross-lagged panel model (CLPM) with the estimation of random intercepts that are allowed to covary (Hamaker et al., 2015). This specification allows for the disaggregation of within- and between-person effects and implicitly controls for stable between-person confounders such as ethnicity, stable aspects of the family environment or stable genetic effects (Speyer et al., 2022). Cross-lagged and autoregressive paths are then defined between the residuals reflecting deviations from the person-specific means and thus allow for insights into within-person processes (Hamaker et al., 2015). Given previous observations of gender differences in many of the constructs under study (Murray et al., 2022), to adjust for the effect of gender, gender was regressed on the random intercepts. We further included second-order cross-lagged effects from prosociality at age 11 to aggressive behaviors and bullying perpetration at age 15 to test for longitudinal mediation. To assess statistical significance of indirect effects (calculated as the product of within-person cross-lagged effects from prosociality to peer relationships and from peer-relationships to aggression/bullying perpetration), we computed standard error using the delta method. However, the delta method assumes a symmetric sampling distribution of indirect effects (MacKinnon et al., 1995) which is unlikely to hold, therefore, we also computed bootstrapped 95% confidence intervals to assess statistical significance.

Given that teacher- and self-reports on children’s psychosocial functioning tend to show limited convergence (De Los Reyes et al., 2015), including in the current sample (Murray et al., 2022), models were fit using either teacher-reported data or self-reported data on aggressive behaviors and prosociality. Two models were fit for each teacher- and self-reported data, one including prosociality, peer relationships and aggressive behaviors, and one including prosociality, peer relationships and bullying perpetration. Given the highly interconnected nature of aggressive behaviors and bullying perpetration, we fitted both separate models for each construct as well as a combined model including both aggressive behaviors and bullying perpetration to provide a more comprehensive understanding of the associations between these constructs and prosociality. Within a combined model, any shared variance between the two behaviors is accounted for, thus, associations give insights into the unique effects of the respective construct on prosociality and vice versa. In contrast, separate models can give insights into the overall effect that aggressive behaviors and bullying perpetration may have on prosociality and vice versa (i.e. their shared as well as unique effect).

Model fit was judged to be good if Tucker Lewis Index (TLI) was >.90, Comparative Fit Index (CFI) was >.90 and Root Mean Squared Error of Approximation (RMSEA) was <.05 (Hair et al., 2010). The RI-CLPMs were fit using Mplus 8.5 (Muthén & Muthén, 1998-2017) using a robust maximum likelihood estimator (MLR) which handles missing data using full information maximum likelihood (Enders, 2001). Mplus code and full model results are provided in the Supplementary Materials Appendixes A-J and on the Open Science Framework (OSF): https://osf.io/76f82/?view_only=18a03271c7b74ddbb1593756aeb22eea.

## Results

### Aggression Model Using Teacher-Reported Data on Prosociality and Aggressive Behaviors

**Figure 1.**
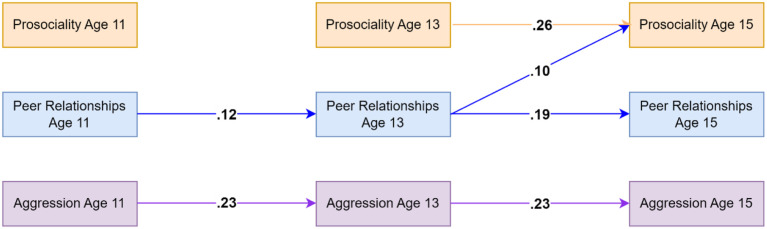
Standardised autoregressive and cross-lagged parameters for the model based on teacher-reported data on prosociality (covering only prosocial behaviors) and aggressive behaviors as well as self-reported data on peer relationships. Only statistically significant paths are shown. Random intercepts and (residual) covariance parameters are omitted for clarity.

Model fit indices indicated good fit (CFI = .986, TLI = .920, RMSEA = .038). Peer relationships and aggressive behaviors showed homotypic continuity across all lags at the within-person level, indicating that, for example, higher levels of aggressive behaviors were associated with higher levels of aggressive behaviors at the next time point relative to individuals' average levels of aggressive behaviors. Prosociality (capturing only prosocial behaviors) was only significantly associated with future increases in prosociality from age 13 to age 15. For cross-lagged effects, results indicated that peer relationships at age 13 were associated with increased prosociality at age 15. Analyses of indirect effects did not suggest evidence for peer relationships mediating the associations between prosociality and aggressive behaviors. Significant standardised autoregressive and cross-lagged parameters are visualized in Figure 1. Residual correlations indicated that aggressive behaviors and prosociality shared moderate, negative within-person associations at ages 11 and 13 (see Table 2(a)). Full results are available on the OSF: https://osf.io/76f82/?view_only=18a03271c7b74ddbb1593756aeb22eea and in the Supplementary Materials Appendixes A and B.

**Table 2.** Residual Correlations.

	Age 11			Age 13			Age 15		
a) Teacher-reported model Aggression	1	2	3	1	2	3	1	2	3
1. Prosociality	-			-			-		
2. Aggression	−.19*	-		−.20*	-		−.07	-	
3. Peer relationships	.13*	−.11*	-	.06	−.08	-	.11*	−.03	-
b) teacher-reported model bullying perpetration	1	2	3	1	2	3	1	2	3
1. Prosociality	-			-			-		
2. Bullying perpetration	−.10	-		−.14*	-		−.13*	-	
3. Peer relationships	.13*	−.20*	-	.07	−.15*	-	.11*	−.07	-
c) self-reported model Aggression	1	2	3	1	2	3	1	2	3
1. Prosociality	-			-			-		
2. Aggression	−.21*	-		−.20*	-		−.07	-	
3. Peer relationships	.23*	−.24*	-	.17*	−.19*	-	.15*	−.11*	-
d) self-reported model bullying perpetration	1	2	3	1	2	3	1	2	3
1. Prosociality	-			-			-		
2. Bullying perpetration	−.16*	-		−.11*	-		−.10*	-	
3. Peer relationships	.25*	−.21*	-	.20*	−.15*	-	.17*	−.07	-

*Note.* *significant at *p* < .05.

### Bullying Perpetration Model Using Teacher-Reported Data on Prosociality

**Figure 2.**
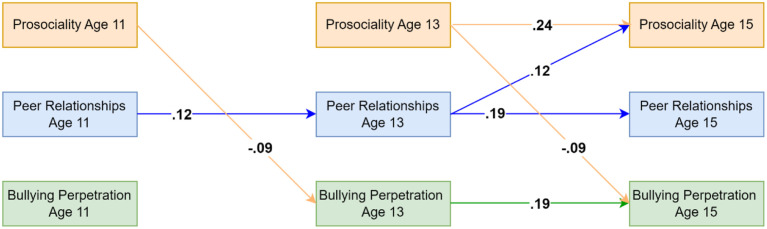
Standardised autoregressive and cross-lagged parameters for the model based on teacher-reported data on prosociality (covering only prosocial behaviors) as well as self-reported data on peer relationships and bullying perpetration. Only statistically significant paths are shown. Random intercepts and (residual) covariance parameters are omitted for clarity.

The model including teacher-reported prosociality (capturing only prosocial behaviors) and self-reported bullying perpetration and peer relationships showed good fit (CFI = .991, TLI = .949, RMSEA = .031). Results indicated that both prosociality and bullying perpetration showed homotypic continuity across the age 13 to 15 lag while peer relationships showed homotypic continuity across ages 11 to 13 and 13 to 15. Across both lags, prosociality was associated with decreases in bullying perpetration at the next time point. No significant mediating effect of peer relationships was identified. For significant standardised autoregressive and cross-lagged parameters, see Figure 2. Residual correlations (available in Table 2(b)) suggested that bullying perpetration and prosociality shared moderate within-person associations at ages 13 and 15. For full results, see the OSF: https://osf.io/76f82/?view_only=18a03271c7b74ddbb1593756aeb22eea and in the Supplementary Materials Appendixes C and D.

### Combined Model of Bullying Perpetration and Aggressive Behaviors Using Teacher-Reported Data on Prosociality

**Figure 3.**
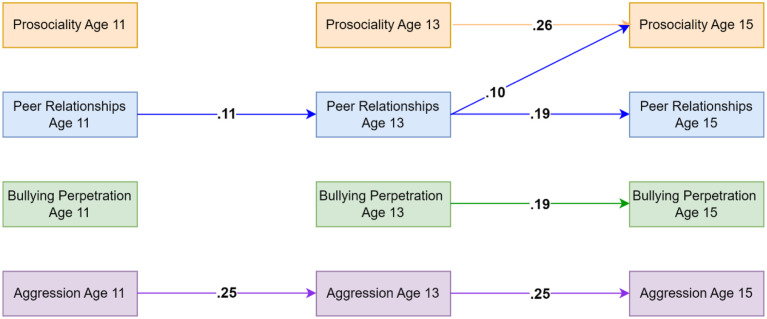
Standardised autoregressive and cross-lagged parameters for the model based on teacher-reported data on prosociality (covering only prosocial behaviors) and aggressive behaviors as well as self-reported data on peer relationships and bullying perpetration. Only statistically significant paths are shown. Random intercepts and (residual) covariance parameters are omitted for clarity.

The model including both aggressive behaviors as well as bullying perpetration also fit the data well (CFI = .988, TLI = .935, RMSEA = .018). Except for the cross-lagged effects from prosociality to decreased bullying perpetration no longer being significant, this combined model was identical to the separate models. Significant standardised parameters are visualized in Figure 3. For full results, please see the OSF: https://osf.io/76f82/?view_only=18a03271c7b74ddbb1593756aeb22eea and Supplementary Appendix E.

### Aggression Model Using Self-Reported Data on Aggressive Behaviors and Prosociality

**Figure 4.**
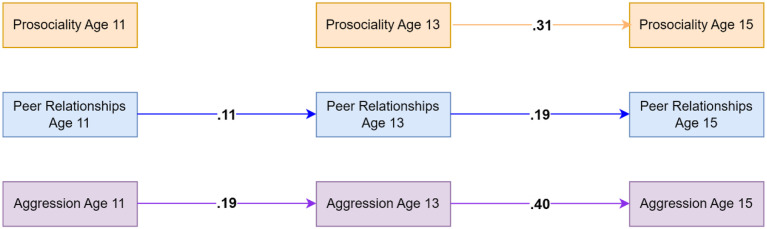
Standardised autoregressive and cross-lagged parameters for the model based on self-reported data on prosociality, aggressive behaviors and peer relationships. Only statistically significant paths are shown. Random intercepts and (residual) covariance parameters are omitted for clarity.

The model using self-reported data on aggressive behaviors and prosociality (capturing empathy, sympathy and prosocial behaviors) also showed excellent fit with CFI = .992, TLI = .953, and RMSEA = .035. Aggressive behaviors and peer relationships showed homotypic continuity across all lags while, prosociality only showed homotypic continuity across the age 13 to 15 lag. No significant cross-lagged or mediating effects were identified. Figure 4 visualizes significant standardised parameters. Residual correlations indicated that aggressive behaviors and prosociality shared moderate within-person associations at ages 11 and 13 (see Table 2(c)). For full results, see the OSF: https://osf.io/76f82/?view_only=18a03271c7b74ddbb1593756aeb22eea and in the Supplementary Materials Appendixes F and G.

### Bullying Perpetration Model Using Self-Reported Data on Prosociality

**Figure 5.**
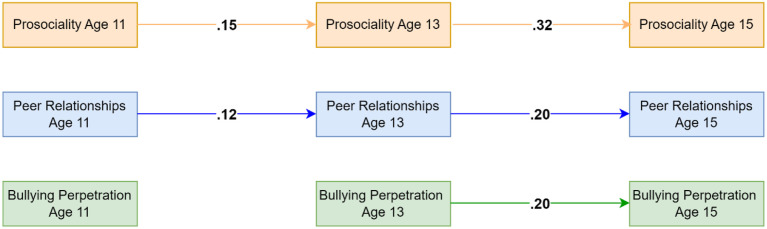
Standardised autoregressive and cross-lagged parameters for the model based on self-reported data on prosociality, bullying perpetration and peer relationships. Only statistically significant paths are shown. Random intercepts and (residual) covariance parameters are omitted for clarity.

This model also showed good fit (CFI = .989, TLI = .943, RMSEA = .037) and showed essentially the same results as the model including self-reported aggressive behaviors. Results suggested an additional autoregressive path from prosociality at age 11 to prosociality at age 13. In contrast to the teacher-reported model on bullying perpetration, the model did not identify any significant cross-lagged effects of self-reported prosociality on bullying perpetration. Analyses of indirect effects also did not find evidence for peer relationships mediating the associations between prosociality and aggressive behaviors. For significant standardised parameter, see Figure 5. Residual correlations suggested that bullying perpetration and prosociality shared moderate within-person associations at all ages (see Table 2(d)). Full results are available on the OSF: https://osf.io/76f82/?view_only=18a03271c7b74ddbb1593756aeb22eea and in the Supplementary Materials Appendix H and I.

### Combined Model For Bullying Perpetration and Aggressive Behaviors Using Self-Reported Data on Prosociality

**Figure 6.**
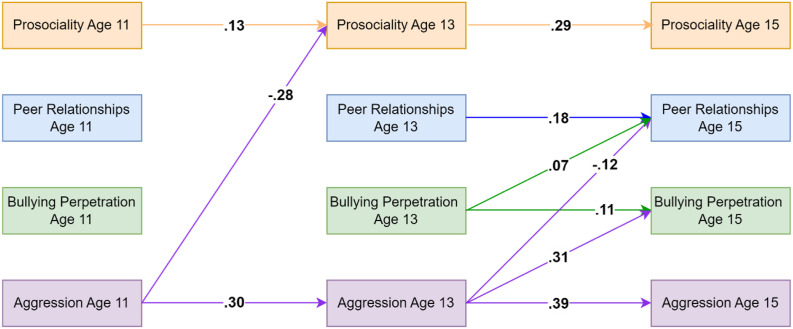
Standardised autoregressive and cross-lagged parameters for the model based on self-reported data on prosociality, bullying perpetration, aggressive behaviors and peer relationships. Only statistically significant paths are shown. Random intercepts and (residual) covariance parameters are omitted for clarity.

The model including both aggressive behaviors as well as bullying perpetration also fit the data well (CFI = .993, TLI = .962, RMSEA = .031). This combined model suggested additional paths from aggression at age 11 being associated with decreases in prosociality at age 13, as well as aggression at age 13 being associated with worse peer relationships at age 15. Further, it suggested that aggression at age 13 was associated with increases in bullying perpetration at age 15, whereas bullying perpetration at age 13 was associated with better peer relationships at age 15. Significant standardised parameters are visualized in Figure 6. Full results are available on OSF: https://osf.io/76f82/?view_only=18a03271c7b74ddbb1593756aeb22eea and in Appendix J.

## Discussion

The aim of the present study was to advance current understandings of the within-person developmental links between prosociality and antisocial behaviors, focusing on whether higher teacher-reported prosociality (capturing only prosocial behaviors) as well as self-reported prosociality (capturing prosocial behaviors, sympathy and empathy) during early adolescence may protect against the development of later aggressive behaviors and bullying perpetration. Further, we investigated whether peer relationships mediate the associations between prosociality and aggressive behaviors as well as bullying perpetration.

We hypothesized that within-person increases in prosociality would be associated with subsequent decreases in aggressive behaviors and bullying perpetration. Results of the teacher-reported models fit separately for bullying perpetration and aggressive behaviors suggested that this hypothesis was partially supported. Specifically, when aggressive behaviors were excluded from the model, teacher-reported prosociality (capturing only prosocial behaviors) protected against engaging in bullying perpetration during early adolescence. However, when bullying perpetration was excluded from the model, higher prosociality was not associated with reduced aggressive behaviors nor were either of these behaviors associated with prosociality when both constructs were included within the same model or when using self-reported data on prosociality.

We further hypothesized that peer relationships would mediate the associations between prosociality and aggressive behaviors as well as bullying perpetration. This hypothesis was not supported. However, we found that having better peer relationships at age 13 was associated with within-person increases in teacher-reported prosocial behaviors at age 15. In addition, results suggested that prosociality and aggressive behaviors as well as prosociality and bullying perpetration are negatively correlated at the within-person level at age 11 and age 13, suggesting that high levels of prosociality are generally associated with less engagement in aggressive behaviors and bullying perpetration concurrently. For bullying perpetration, this was also found at age 15.

One potential reason for why teacher-reported prosociality may protect against future engagement in bullying perpetration could be that engaging in prosocial behaviors gives young people positive feedback in that it feels good to be nice to people, that is resulting in some form of social reward. Within framing theory as outlined in the introduction (Veenstra, 2006), this would suggest that adolescents may refrain from bullying perpetration as they act following a gain frame, having realised that prosociality leads to higher social reward then engaging in antisocial behaviors. This would also suggest that prosociality would lead to better peer relationships which may in turn protect against engaging in bullying perpetration, however this was not found in the current study. It is possible that this was due to the time-frame under investigations having been too long to observe such a mechanism. Future research could explore this in more detail, for instance using ecological momentary assessment studies that capture the processes in daily life that relate to prosocial behavior (e.g., does it lead to more positive affective states), or using qualitative interviews to illuminate this.

Results further suggest that monitoring children’s prosocial behavior may be beneficial for identifying children at risk of the development of antisocial behaviors, particularly since teacher-reported prosociality was associated with bullying perpetration whereas self-reported prosociality was not. Importantly, teachers and parents should not only attend to overall levels of prosociality but also whether an adolescent deviates from their usual levels since decreases in prosociality from a young person’s norm may be a particularly strong marker for an increased risk of later engagement in antisocial behaviors.

The fact that the observed direct protective effect of prosociality on the development of bullying perpetration was only observed in the teacher-reported model further suggests that prosociality may have context-specific effects. Specifically, aspects of prosociality that can be observed in the classroom, that is, are more behavior based, may be particularly important when it comes to protecting against antisocial behaviors that tend to occur in the school context such as bullying behaviors. Self-reports on prosociality as included in the current study were less context-specific as individuals reported on their prosociality across a much larger range of contexts than teachers, including reporting on their internal states such as feelings of empathy and sympathy, suggesting that more general prosociality reported by young people themselves may not directly translate to problematic behaviors that tend to occur in the school-context such as bullying perpetration. However, re-running the bullying perpetration model including only the self-reported prosocial behaviors items showed the same results, that is, no association of prosocial behaviors with future engagement in bullying perpetration (see results in and in the Supplementary Materials Appendix K and L).

Discrepancies in teacher-compared to self-reports have been frequently found in the literature (e.g., Murray et al., 2022), thus, highlighting the importance of investigating questions such as those posed in the current study using a multi-informant perspective. One other potential mechanism driving the association between teacher-reported prosociality and bullying perpetration could be a labelling effect. Specifically, a teacher’s assessment of a child not being very prosocial may lead to increases in the child’s antisocial behaviors due to reactions of the child and others to negative stereotypes that are associated with being labelled to be less prosocial (Bernburg, 2009). Interestingly, in neither the self- nor teacher-reported models did we find evidence for a direct protective effect of prosociality on aggressive behaviors. This is consistent with findings by Obsuth et al. (2015) for the same association during childhood. The lack of effects could potentially be due to the more commonplace nature of some of the bullying items reflecting milder behaviors, for instance relating to ignoring someone, compared to hitting someone. We also found a negative path from aggressive behaviours at age 11 to prosociality at age 13 in the combined self-reported model (i.e., the model including both aggressive behaviours and bullying at the same time), but this path could not be found from age 13 to age 15 and was not replicated in the models that examined effects for bullying and aggression separately.

Interestingly, this contrasts with previous findings from the same sample during a younger age range, spanning ages 7 to 11 (Obsuth et al., 2015). In that study, Obsuth et al. found that teacher-as well as parent-reported aggressive behaviors measured one year apart were linked to a reduction in prosocial behaviors the following year across ages 7 to 11. For self-reported data, such an effect was only observed from age 7 to 8. In contrast to our study, Obsuth et al. did not find evidence for effects in the opposite direction neither for self- nor teacher-reported data. Considering the results of both Obsuth et al. (2015) and the current study, findings suggest potential developmental differences in the associations between prosocial and antisocial behaviors, with prosocial behaviours only emerging as a protective factor for some antisocial behaviors during early adolescence. Additionally, findings suggest that aggressive behaviors in middle childhood could represent a more longer term risk factor for young individuals’ social development. Specifically, cascading effects starting in childhood may indirectly extend into adolescence. As shown in Obsuth et al. (2015) aggressive behaviors in childhood were associated with a heightened risk of exhibiting lower prosocial behavior in late childhood (up until age 11), which in turn, as shown in the current study, might lead to a subsequent increase in engaging in bullying perpetration. This suggests that interventions promoting prosocial behaviors in early-to-mid-adolescence may be beneficial for reducing bullying across development and that, reflecting on Obsuth et al.’s (2015) findings, children displaying aggressive behaviors in childhood may particularly benefit from such support.

Another potential reason for the difference in findings between the current study and previous research into the links between prosocial and antisocial behaviors is worth acknowledging. The majority of prior studies, including the aforementioned research by Obsuth et al. (2015), have examined similar research questions using statistical designs that do not differentiate within-person and between-person effects. Hence, it may also be that previously observed links between aggressive behaviors and prosociality are related to between-person differences rather than to processes unfolding at the within-person level. Future studies, including replication studies using the same statistical designs, are necessary to untangle the associations between prosociality and antisocial behaviors across development further.

Our results can also inform debates as to whether prosociality is purely the opposite of antisociality. Results of the raw associations between prosociality and bullying perpetration as well as aggressive behaviors provide further evidence that this is not the case. Specifically, if these two constructs were indeed two sides of the same coin, we would have observed strong and consistent associations between prosociality and bullying perpetration as well as aggressive behaviors across all time-points. However, we found that constructs were only weakly correlated, ranging from −.12 to −.32 for prosociality and aggressive behaviors, and from −.14 to .24 for prosociality and bullying perpetration (correlations are reported in full in Table S3 in the online Supplementary Materials). Further, we found that raw correlations between prosociality and aggressive behaviors decreased across development, whereas associations with bullying perpetration tended to show an increase in the strength of associations. These differential associations between prosociality and bullying perpetration and aggressive behaviors across early-to mid-adolescence further suggest that prosociality and antisociality may not just be opposites of each other. Future research is needed to disentangle the respective mechanisms that link prosociality to different types of antisocial behaviors across development.

Overall, results of this study suggest that, following a risk and protective factor framework, prosociality may act as a direct protective factor (‘promotive’ factor following Farrington et al.’s (2016) definition) for reducing some forms of antisocial behaviors, that is, specifically bullying perpetration. Within a risk-need-responsivity framework (Bonta & Andrews, 2007), which aims to help identify individuals at risk of engaging in antisocial behaviors as well as identify treatment targets, results of the current study suggest that prosociality may be a vital component when assessing an individual’s risk of future engagement in antisocial behaviors. As such, prosociality may be an important factor to consider in interventions aiming to prevent or reduce such behaviors. Specifically, school-based bullying prevention programs may benefit from including components that support enhancing prosociality. Recommended whole-school anti-bullying approaches could easily incorporate such components. For example, the KiVa anti-bullying program, one of the most supported-by-evidence anti-bullying approaches focuses on improving the whole-school culture by targeting children as well as teachers and parents. Through a combination of universal, that is preventative actions, and indicative actions in the event of bullying incidents, KiVa covers a variety of topics related to bullying, including group dynamics and how they can promote or prevent bullying as well as raising awareness for bullying and its consequences more generally (Salmivalli & Poskiparta, 2012). Adding a component on practicing prosocial behaviors specifically, for example through role play, as well as raising awareness of prosociality and how it can positively influence, for instance group dynamics, could be incorporated into existing programs like KiVa and potentially increase its efficacy.

In the context of the current study, it is important to note that z-proso also included a randomized control trial examining the impact of two intervention programs (Promoting Alternative Thinking Strategies (PATHS) and the Triple-P (Positive Parenting Program)) aiming to reduce externalising behaviors (Malti et al., 2011). The PATHS program focused on social skills training and promoted, among others, prosociality and empathic skills. However, results of the randomized control trial did not find any longer term beneficial effects of this program on adolescents’ externalising behaviors (Averdijk et al., 2016), thus, further research refining such intervention programs is needed to improve their efficacy.

While this study has a number of strengths including its use of a longitudinal cohort spanning the age range of early to middle adolescence as well as the use of an analysis design that allows within-person effects to be isolated from between-person effects, there are a number of limitations that need to be taken into consideration. First, the time lags between waves may not have been optimal for capturing the relations between these constructs (Dormann & Griffin, 2015; Eisner & Malti, 2015). For instance, it may be that prosociality is associated with reduced aggressive behaviors over shorter lags such as a few months rather than the here investigated associations spanning time lags of two years. The fact that within-person concurrent residual associations were significant at most time-points supports this as these associations suggest that concurrent high levels of prosociality are associated with less engagement in antisocial behaviors at the same time-point. Interestingly, we found that aggressive behaviors were only concurrently associated with prosociality at ages 11 and 13 but not at age 15. This suggests that there may be developmental differences in how prosociality relates to aggressive behaviors in that prosociality does not relate as closely to aggressive behaviors during mid-adolescence compared to early adolescence. This could be due to developmental changes in the type of aggressive behaviors that may be more common across later stages of adolescence, for instance relating more to relational compared to more direct forms of aggression (see Voulgaridou & Kokkinos, 2015 for a review on relational aggression during adolescence). This is also supported by the fact that bullying perpetration shared concurrent associations with teacher-reported prosociality at age 15 but not with teacher-reported prosociality at age 11. Indeed, analyses on developmental invariance of the bullying items used in the current study have previously suggested that bullying items focusing on physical acts of bullying become a poorer marker of overall bullying across adolescence (Murray et al., 2021), thus further suggesting that different forms of antisocial behavior become more or less salient over time.

In this context, it also needs to be acknowledged that teacher- and self-reported data were not measured concurrently. While the reference time-frames generally aligned well between self-report and teacher assessments, the latter were often completed weeks or months after the self-report assessments, potentially influencing findings. Furthermore, although most measures required individuals to reflect on their experiences over the past year, responses regarding peer relationships referred to their current classroom situation, potentially encompassing a distinct time-frame from items concerning aggressive or prosocial behaviors. This could introduce an additional confounding factor, particularly concerning the likely sequence of events over time.

Further, the brevity of some of the included measures may have limited their ability to capture the full spectrum of behaviors, thus reducing their content validity. Moreover, brief measures are more prone to random measurement error which could have attenuated potential associations between the constructs under investigation. Also, we were not able to investigate the target of the behaviors under investigation, that is family members, friends, or strangers. Prior research has suggested that antisocial as well as prosocial behaviors may serve different functions and may be differently associated with other outcomes depending on the target that such behaviors are primarily directed towards (Padilla-Walker et al., 2015). Future research is needed to disentangle these differential effects further. Similarly, future research may benefit from explicitly testing the role of antisocial versus prosocial peers in the association between antisocial behaviors and prosociality. With regards to the measure of bullying perpetration, prior evidence has suggested that bully-victims are the highest risk group for developing further emotional and behavioral issues, however, we were not able to investigate whether being a bully victim may moderate the associations that we observed in relation to bullying perpetration. Further, the ZBBS does not fully capture the complexity of power imbalances that are integral to the concept of bullying (Craig & Pepler, 2007). Bullying perpetration may have different antecedents and effects depending on the target of the behavior and the power dynamics involved. To better assess this aspect of bullying, future research should incorporate additional measures or items that more effectively capture these nuances.

Another limitation that needs to be acknowledged is that findings derived from one cohort study may not readily generalize to other studies, owing to the distinctive social and cultural contexts inherent to each cohort. For example, the transition from attending comprehensive primary school to a tiered system of secondary schools by educational achievement at age 12/13 may have affected peer relationships, and the school contexts for both prosocial and aggressive behavior. For more insights into the context of the z-proso study, please refer to the introductory paper of this special issue (REFERENCE TO BE ADDED BY JOURNAL).

Of importance to note is also that observed effect sizes were relatively small (ranging from .09 to .18 for cross-lagged effects in the separate models and from .07 to .31 for cross-lagged effects in the combined models); however, in the context of longitudinal models accounting for stability effects, effect sizes are expected to be small and may still represent valuable intervention targets, especially since they accumulate over time (Adachi & Willoughby, 2014).

With regards to the statistical approach used in the current study, that is Random Intercept Cross-Lagged Panel Models, due to the temporal nature of effects, coefficients from such models are often implicitly taken to represent causal effects and as such interpreted. However, we note that such models are a limited tool for causal inference (see e.g., Muthén & Asparouhov, 2023). For examples, results can be sensitive to variations in model specifications. In the current study, results that considered bullying perpetration and aggressive behaviours separately did mostly not replicate when both behaviors were considered in the same model. This may reflect differences in how the different models capture the unique versus the total effect of the constructs under investigation. More broadly, results of RI-CLPMs may also be affected by unmeasured confounders, between-informant differences in the timing of data collection, and a lack of consolidated knowledge about the correct lag structure of putative causal effects which all limit our ability to draw causal conclusions based on the results of a single study. Like any statistical model, RI-CLPMs are limited in capturing the complexities of human behavior. Consequently, conclusions on causal structures will need to be drawn with caution and ideally across multiple models and multiple datasets.

## Conclusion

In conclusion, results of the current study suggest that increases in teacher-reported prosociality during early-to mid-adolescence may protect against the development of some antisocial behaviors, specifically bullying perpetration. These findings suggest that intervention programs aiming to prevent the development of antisocial behaviors, such as anti-bullying programs, may benefit from incorporating components specific to promoting prosociality.

## Supplemental Material

Supplemental Material - Does Prosociality in Early-to Mid-Adolescence Protect Against Later Development of Antisocial Behaviours? Supplemental Material for Does Prosociality in Early-to Mid-Adolescence Protect Against Later Development of Antisocial Behaviours? by Lydia Gabriela Speyer, Ingrid Obsuth, Manuel Eisner, Denis Ribeaud, and Aja Louise Murray in The Journal of Early Adolescence

Supplemental Material - Does Prosociality in Early-to Mid-Adolescence Protect Against Later Development of Antisocial Behaviours? Supplemental Material for Does Prosociality in Early-to Mid-Adolescence Protect Against Later Development of Antisocial Behaviours? by Lydia Gabriela Speyer, Ingrid Obsuth, Manuel Eisner, Denis Ribeaud, and Aja Louise Murray in The Journal of Early Adolescence
