# Supplementary material for: Does Prosociality in Early-to Mid-Adolescence Protect Against Later Development of Antisocial Behaviours?
Source: J Early Adolesc. 2023 Nov 13;44(9):1124–53. doi: 10.1177/02724316231210254 (PMC11446672; doi:10.1177/02724316231210254)
Supplement: Supplemental Material - Does Prosociality in Early-to Mid-Adolescence Protect Against Later Development of Antisocial Behaviours? [file sj-pdf-1-jea-10.1177_02724316231210254.pdf]

Supplementary Materials

Table S1. Questionnaire items

**Self-reported prosociality items**
You volunteered to help to tidy or clear up a mess.
You were good at understanding another person’s feelings.
You shared your own things with others.
When others had a quarrel or dispute you tried to stop it.
You tried to help someone who had hurt himself/herself.
You tried to comfort someone who was crying or who was upset.
You listened carefully to the point of view of others.
You showed sympathy to someone who was upset or had hurt himself/herself.
**Self-reported aggressive behaviours items**
You hit or kicked your parents when you were angry.
You got very angry when someone teased or irritated you.
When you were mad at another kid you said bad things about him/her behind the kid’s back.
You intimidated someone else to get what you wanted.
You physically attacked other people.
You ordered others around.
When you were mad at another kid you got others to dislike that kid as well.
You kicked, bit, or hit someone else.
You yelled at your parents in anger.
When you were mad at another kid you said to others: “Let’s not be with him/her”.
You hit someone when they tried to take something from you.
You threatened others so that they would give you something.
You threw an object at your parents in anger.
You got into fights.
You “went ballistic” when you didn’t get what you wanted.
**Teacher-reported prosociality items**
<CHILD> volunteers to help clear up a mess someone else has made.

If there is a quarrel or dispute, <CHILD> will try to stop it.

<CHILD> will try to help someone who has been hurt.

<CHILD> spontaneously helps to pick up objects, which another child has dropped (e.g. pencils, books, etc.).

<CHILD> comforts a child who is crying or upset.

<CHILD> shares things with others.

Teacher-reported aggressive behaviours items

<CHILD> gets into fights.

<CHILD> physically attacks people.

<CHILD> kicks, bites, hits other children.

<CHILD> is cruel, bullies or is mean to others.

<CHILD> threatens people.

<CHILD> encourages other children to pick on a particular child.

<CHILD> tries to dominate other children.

<CHILD> scares other children to get what he\she wanted.

<CHILD> reacts in an aggressive manner when teased.

<CHILD> reacts in an aggressive manner when something was taken.

<CHILD> reacts in an aggressive manner when contradicted.

<CHILD> systematically excludes others.

<CHILD> insults/humiliates others.

Self-reported peer relationships in the classroom

We have a really good sense of community within the class.

I get on well with the other kids/adolescents in my class.

The other kids/adolescents in my class are nice to me.

Self-reported bullying perpetration

How many times have you purposely ignored or excluded another kid?

How many times have you laughed at, mocked, or insulted another kid?

How many times have you hit, bitten or kicked another kid, or pulled their hair.

How many times have you purposely stolen, broken or hidden another kid’s things?

Table S2. Descriptive Statistics

**Variable**	***n***	***mean***	***sd***	***min***	***max***	***range***	***skew***	***kurtosis***	***reliability***
Self-reported prosociality age 11	1106	3.733	0.678	1.375	5.000	3.625	-0.456	0.141	.91
Self-reported prosociality age 13	1298	3.562	0.682	1.000	5.000	4.000	-0.399	0.036	.92
Self-reported prosociality age 15	1399	3.605	0.632	1.250	5.000	3.750	-0.318	0.160	.89
Self-reported aggressive behaviours age 11	1078	1.468	0.386	1.000	3.533	2.533	1.576	3.478	.95
Self-reported aggressive behaviours age 13	1277	1.727	0.500	1.000	4.800	3.800	1.303	2.685	.95
Self-reported aggressive behaviours age 15	1375	1.676	0.473	1.000	4.000	3.000	1.326	2.457	.94
Peer relationships age 11	1141	3.338	0.603	1.000	4.000	3.000	-0.840	0.518	.87
Peer relationships age 13	1361	3.291	0.615	1.000	4.000	3.000	-0.801	0.609	.77
Peer relationships age 15	1445	3.290	0.599	1.000	4.000	3.000	-0.745	0.486	.82
Bullying perpetration age 11	1144	1.541	0.661	1.000	5.000	4.000	1.953	4.846	.75
Bullying perpetration age 13	1363	1.722	0.765	1.000	5.500	4.500	1.602	2.954	.78
Bullying perpetration age 15	1443	1.766	0.763	1.000	6.000	5.000	1.634	3.566	.75
Teacher-reported prosociality age 11	1028	3.254	0.874	1.000	5.000	4.000	-0.117	-0.442	.79
Teacher-reported prosociality age 13	1129	3.085	0.859	1.000	5.000	4.000	-0.115	-0.294	.82
Teacher-reported prosociality age 15	1151	3.077	0.815	1.000	5.000	4.000	-0.026	-0.242	.79
Teacher-reported aggressive behaviours age 11	1040	1.480	0.621	1.000	5.000	4.000	1.882	3.923	.82
Teacher-reported aggressive behaviours age 13	1210	1.355	0.541	1.000	5.000	4.000	2.444	7.621	.86
Teacher-reported aggressive behaviours age 15	1227	1.336	0.490	1.000	5.000	4.000	2.251	6.261	.85

Notes. Reliability = Cronbach’s alpha.

Table S3. Raw Correlations

**Teacher-reported data on prosociality and aggressive behaviours**	1.	2.	3.	4.	5	6.	7.	8.	9.
1. Prosociality Age 11	-								
2. Prosociality Age 13	.23	-							
3. Prosociality Age 15	.22	.44	-						
4. Aggressive Behaviours Age 11	-.32	-.17	-.19	-					
5. Aggressive Behaviours Age 13	-.18	-.32	-.25	.41	-				
6. Aggressive Behaviours Age 15	-.10	-.18	-.26	.28	.40	-			
7. Bullying Perpetration Age 11	-.14	-.06	-.08	.21	.10	.13	-		
8. Bullying Perpetration Age 13	-.18	-.17	-.15	.15	.15	.14	.35	-	
9. Bullying Perpetration Age 15	-.15	-.18	-.21	.16	.20	.26	.37	.45	-

**Self-reported data on prosociality and aggressive behaviours**	1.	2.	3.	4.	5	6.	7.	8.	9.
1. Prosociality age 11	-								
2. Prosociality Age 13	.47	-							
3. Prosociality Age 15	.42	.57	-						
4. Aggressive Behaviours Age 11	-.25	-.22	-.13	-					
5. Aggressive Behaviours Age 13	-.17	-.27	-.15	.44	-				
6. Aggressive Behaviours Age 15	-.10	-.15	-.12	.36	.57	-			
7. Bullying Perpetration Age 11	-.24	-.15	-.09	.60	.28	.21	-		
8. Bullying Perpetration Age 13	-.14	-.24	-.12	.36	.59	.36	.35	-	
9. Bullying Perpetration Age 15	-.19	-.23	-.21	.35	.46	.57	.35	.45	-
